# An effective and efficient approach for manually improving geocoded data

**DOI:** 10.1186/1476-072X-7-60

**Published:** 2008-11-26

**Authors:** Daniel W Goldberg, John P Wilson, Craig A Knoblock, Beate Ritz, Myles G Cockburn

**Affiliations:** 1 Department of Computer Science, University of Southern California, Los Angeles, CA, USA; 2 Department of Geography, University of Southern California, Los Angeles, CA, USA; 3 Information Sciences Institute, University of Southern California, Marina del Rey, CA, USA; 4 Department of Epidemiology, University of California, Los Angeles, Schools of Public Health and Medicine, Los Angeles, CA, USA; 5 Department of Preventive Medicine, University of Southern California, Los Angeles, CA, USA

## Abstract

**Background:**

The process of geocoding produces output coordinates of varying degrees of quality. Previous studies have revealed that simply excluding records with low-quality geocodes from analysis can introduce significant bias, but depending on the number and severity of the inaccuracies, their inclusion may also lead to bias. Little quantitative research has been presented on the cost and/or effectiveness of correcting geocodes through manual interactive processes, so the most cost effective methods for improving geocoded data are unclear. The present work investigates the time and effort required to correct geocodes contained in five health-related datasets that represent examples of data commonly used in Health GIS.

**Results:**

Geocode correction was attempted on five health-related datasets containing a total of 22,317 records. The complete processing of these data took 11.4 weeks (427 hours), averaging 69 seconds of processing time per record. Overall, the geocodes associated with 12,280 (55%) of records were successfully improved, taking 95 seconds of processing time per corrected record on average across all five datasets. Geocode correction improved the overall match rate (the number of successful matches out of the total attempted) from 79.3 to 95%. The spatial shift between the location of original successfully matched geocodes and their corrected improved counterparts averaged 9.9 km per corrected record. After geocode correction the number of city and USPS ZIP code accuracy geocodes were reduced from 10,959 and 1,031 to 6,284 and 200, respectively, while the number of building centroid accuracy geocodes increased from 0 to 2,261.

**Conclusion:**

The results indicate that manual geocode correction using a web-based interactive approach is a feasible and cost effective method for improving the quality of geocoded data. The level of effort required varies depending on the type of data geocoded. These results can be used to choose between data improvement options (e.g., manual intervention, pseudocoding/geo-imputation, field GPS readings).

## Background

Geocoded data, which are geographic representations of textual location descriptions such as postal addresses, are used routinely throughout health-related research. The literature regarding the process of geocoding, the accuracy of the geocodes produced, and the effects of varying geocode accuracy are well documented [1-7]. It is widely acknowledged that (1) all geocoded points do not represent locations with the same level of certainty; (2) the variation in certainty within datasets of geocodes is most likely not random; and (3) simply excluding cases that are non-geocodable or geocodable to a low level of accuracy should not, under most circumstances, be considered an acceptable practice. Without ground truth data, it is impossible to obtain a true "accuracy" value for a geocode which is a spatial measure of the distance between the geocode location and the true location. Therefore when people speak of geocode "accuracy", they are actually referring to the purported accuracy of the geocode based one or more characteristics about the geocode and/or its production. It is within this context of the notion of "purported accuracy" about the geocode that we will use the term "accuracy".

The geocoding process is extraordinarily complex and the accuracy of the output geocodes can range from representing the center of a roofline to the centroid of a county (see [3,6,7] for reviews of the technical details of the process). Each level of geocoding accuracy (e.g., building centroid, parcel centroid, city centroid) denotes an area of uncertainty with which the location could be associated [8]. The literature suggests that USPS ZIP code centroid accuracy may be a cutoff for useful data in health studies [4,9-12], although concerns about even this level of accuracy are prevalent [13]. Some studies do report the quality of the geocodes used [6,14-17] and/or how they were determined [18]. But, as noted by [6,17,19,20], many studies still report a single match rate (the number of input addresses successfully geocoded out of the total number of input addresses attempted) as the measure of geocoding quality without indicating anything about the accuracy of the geocodes used, e.g., [21,22], or even any mention of geocode quality at all, e.g., [23,24]. Recent work has provided guidelines to handle varying levels of uncertainty in a geocoded dataset [25,26]. These approaches are applied after the automated geocoding process has been completed and are meant to mitigate the effects of utilizing geocodes of varying qualities within a single study. This approach does not actually improve the accuracy of low quality geocodes. Instead of mitigating geocoding error after the fact, poor quality geocoded and/or non-geocodable data can be reprocessed to achieve a higher quality and/or successful geocoded result. Methods for improving upon automated geocoding processes consist of manual intervention/interactive geocoding [27,28], re-geocoding with a different geocoding strategy [29,30], geographic imputation/pseudocoding [7,10,11], or any combination thereof.

The manual intervention/interactive geocoding process involves checking an input address for obvious errors that can trivially be corrected; such as attribute transposition where portions of the input address have been entered in an incorrect or unexpected order (as described in [18]). An enhancement could involve researchers resolving ambiguous address information with additional knowledge about the address (over and above the street, city, state, USPS ZIP code fields). One example of this is an occupational address reported as "USC", which could either refer to the University of Southern California or University of South Carolina. In this case, knowledge about the study area and/or population of interest could be used to determine the correct university. For records where an address or address component is incorrect or missing, a detailed investigation can be performed to determine an appropriate and/or corrected address to be geocoded based on other information associated with the record. Commonly used sources include websites, phone books, utility records, and various vital records [14,21,31-33]. These processes are typically termed "interactive geocoding", e.g., [31].

Re-geocoding data with a different geocoder is often used for records that were non-geocodable or resulted in low quality geocodes after a first geocoding attempt [29,30]. This strategy presupposes that multiple geocoding options are available to a researcher, and is often employed when there are cost differentials between using them. For instance, if a free geocoder is available that does an adequate job on most data, and another geocoder exists that does a fantastic job for some fee, it would be a reasonable strategy to make a first pass with the free one and submit only the non-geocodable and/or low quality ones to the high cost service. The reverse has also been reported in the literature [28].

Producing an approximation of the true geocode based on additional information has been described as the processes of creating a pseudocode and/or geographic imputation (geo-imputation) [7,10,11,25]. In these processes, characteristics of the population or the geographic region can be used in conjunction with probability distribution functions to compute a plausible location for the output geocode. For in-depth theoretical details on the applicability of these approaches see [25]. Recent work [10] has found that as additional US Census Bureau demographic variables are used to derive location of the output, the resulting geocodes become increasingly more accurate; eventually surpassing the accuracy of using lower-quality geocodes (postal code centroids). However, all pseudocoding options approximate where an output geocode should most likely be, given the information available. Utilizing such methods introduces a certain level of uncertainty into each geocode produced, and it has been stated in the literature that "geo-imputation does not, by any means, fully compensate for low quality geocoding" and that "improving the underlying quality of the address data [and initial geocode] is an ideal alternative to geo-imputation" [10].

It is not clear if it is worthwhile to perform the manual intervention process, and whether the amount of effort involved actually improves the dataset. Pertinent questions are how much improvement can be gained through manual intervention, and how much effort is required to obtain that improvement (i.e., is it worth it)? Although the practice is used throughout the health literature [2,11,14,15,17-19,26-29,34-36] and elsewhere [37-40], little quantitative research exists on the benefits or drawbacks of employing a manual intervention approach for improving the quality of one's geocodes in terms of the effort required. Manual approaches have typically been applied to small scale problems [11,19,26,27,33-36], and/or to samples of addresses representing a population with a single characteristic, e.g., all within a single area [15,26,27,36], all rural addresses [15,26,34], all addresses of individuals with a particular type of cancer e.g., breast cancer [14], or all industrial complexes [35]. Some studies have used the process for medium scale projects in areas with varying characteristics [2]. Gilboa et al. [31] evaluated the match rate improvements obtainable from using an interactive approach, but differs from the present work in that they did not report the effort required other than the anecdotal observation that the process was "time consuming" nor did they report correcting the location of output geocodes through the use of imagery or any other GIS layers.

A thorough review of the literature has revealed only three reports detailing the effort required for this task. The first was outside of the health domain, applying the approach to the creation of a GIS address database for Belo Horizonta, Brazil creating and/or verifying geocodes for 400,000 addresses [41], proving that the approach was feasible for very large datasets. The second was directly applicable to health data, but the manual intervention process was not the central aspect of the research [11] and effort estimates were only offered as secondary considerations. Perhaps most closely related is the work by Boscoe et al. [33]. The research question they investigate is in much the same spirit as that of the present work, and effort estimates are reported. However, the sample size of manually processed records used in their work (n = 500) is much smaller than that attempted herein (n = 22,317). Therefore, it remains difficult for a health researcher to assess how well these approaches will apply or how much effort will be required for different sized health-related datasets and/or samples of address data with non-uniform and/or untested characteristics.

The present work seeks to address this lack of quantitative data by investigating the time and effort required to correct the geocodes contained in five health-related datasets, typical of data commonly used in Health GIS. The current work also addresses the call put forth to the research community by Ward and Wartenberg that epidemiologists will be able to improve the accuracy, precision, and sensitivity of their investigations of the etiology of environmentally caused disease by changing and improving the exposure assessment methodology as new technology and data are made available [[42], pp 210].

To these ends, we developed and used a web-based system for rapid manual intervention of previously geocoded data where we drew from online satellite imagery, street maps, and a secondary geocoding engine to determine an improved geocode for each record [43]. The main functions of the system were: (1) to allow users to interactively research and re-geocode data that failed the initial geocoding to obtain successful geocodes for each record; and (2) to enable users to verify and/or improve the accuracy of previously successfully geocoded records. Users processed records one at a time, and all of the actions they performed for each record were recorded, including how long it took and how/why they corrected a geocode in the fashion they did. Our method makes use of entirely free data so the person-hours were the only costs incurred. The system is free for all researchers , and the complete source code can be made available upon request to researchers and/or organizations that would like to use or improve it.

## Results

We provide below some major observations from the manual geocoding of the 22,317 records, commenting on the time taken to process the records, the improvement in accuracy, and the distance between original and improved results. We define an improvement to a geocode to be one of two cases: 1) when a record was originally non-geocodable and a geocode was obtained after processing; or 2) when a record was previously geocodable and the accuracy of its geocode was improved after processing. It should be noted that we consider a record that has a lower Geocode Quality Code (from Table 1) after it has been processed to be an accuracy improvement according to criteria 2, although without ground truth data it is not possible to quantitatively assess how the accuracy of the original geocode has improved.

**Table 1:** Geocode Quality Codes with corresponding NAACCR GIS Coordinate Quality Codes

Geocode Quality Code	NAACCR GIS Coordinate Quality Code	Description
1	1	Global Positioning System

2	N/A	Building centroid

3	2	Exact parcel centroid

4	2	Nearest parcel centroid

5	3	Actual lot interpolation

6	3	Uniform lot interpolation

7	3	Address range interpolation

8	4	Street intersection

9	5	Street segment mid-point

10	6	ZIP+4

11	7	ZIP+2

12	9	ZIP

13	10	PO Box / Rural Route ZIP

14	11	City centroid

15	N/A	Minor civil division centroid

16	12	County centroid

17	N/A	State centroid

18	N/A	Country centroid

99	99	Non-assignable

For 3,507 (16%) of the records, geocode correction resulted in accuracy improvements according to criteria 1, meaning that they were previously non-geocodable and after processing had geocodes. For 8,773 (39%) of the records, geocode correction resulted in accuracy improvements according to criteria 2, meaning that a geocode with higher accuracy than that of the original geocode was obtained. Overall, 12,280 (55%) of the records were improved using the geocode correction process according to criteria 1) and 2) together.

### Processing time

Geocode correction of five health-related datasets containing a total of 22,317 records was completed in 11.4 weeks (i.e., 427 hours of total of active work). The average processing time per record was 69 seconds when taken across the complete 22,317 record dataset containing records that both could and could not be improved. Of the 12,280 (55%) of records that were improved according to criteria 1 and 2 together, the geocode correction process took 95 seconds per record on average across all five datasets. In the following sections, we will focus the discussion of our results on the records that were able to be improved using our process in order for the reader to assess the value that our approach can add to their data, as compared to the cost in terms of the effort required.

For criteria 1 – when correcting from a previously non-geocodable address – the shortest average correction time was 98 seconds and occurred when obtaining a county level accuracy geocode as the outcome. Note that even though these resulting county level accuracy geocodes improve the overall match rate of the dataset, they may still be of insufficient accuracy to be useful in health studies. The longest average correction time when correcting from a previously non-geocodable address was 449 seconds (7.5 minutes) and occurred when obtaining the nearest parcel centroid accuracy geocode. Spatial shifts comparing the pre- and post-processing locations are not reported for these previously non-geocodable points because, by definition, non-geocodable data do not have pre-processing locations from which to calculate the shift.

For criteria 2 – when an address was previously geocodable and its accuracy was improved – the shortest average correction time was 19.4 seconds and occurred when correcting from county to city level accuracy, resulting in an average spatial shift of 96 km. The longest average correction time for an accuracy improvement was 397 seconds (6.6 minutes) and occurred when correcting from city level accuracy to parcel centroid level accuracy, resulting in a spatial shift of 3.5 km.

### Spatial shifts for criteria 2 improvements

In general, the spatial shift between the location of original batch generated geocodes and their corrected counterparts for criteria 2 improvements averaged 9.9 km per record. The smallest average spatial shift was 32 m and occurred when correcting from nearest parcel centroid to building level accuracy, taking 62 seconds on average. The largest average spatial shift was 110 km and occurred when correcting from USPS ZIP code level accuracy to street centroid level accuracy, taking 149 seconds (2.5 minutes) on average.

### Match rate improvements (criteria 1 improvements)

The match rates before and after the geocoding improvement process are shown for all datasets combined, and by dataset in Table 2, which also lists the number of records that were corrected to a higher level of accuracy. The overall match rate of the five datasets improved from 79.3 to 95%. The match types (i.e., accuracy levels) and number of occurrences before and after the correction process are shown for all datasets combined and by dataset in Table 3. The number of initially unmatched records that were successfully matched through manual intervention are shown in Table 4, broken down by resulting match type.

**Table 2:** Match rates before and after correction and records with improved accuracies

Dataset	Number of Records n	Original Match Rate %: n	Corrected Match Rate %: n	Accuracy Improvements %: n
Hospitals	418	100%: 418	100%: 418	86.1%: 360

Radiation	2,011	99%: 1,990	100%: 2,010	92.7%: 1,865

PD	17,471	81.6%: 14,252	97.4%: 17,013	34.7%: 6,064

Prostate Res.	1,944	53.5%: 1,309	71.7%: 1,393	24.9%: 484

Prostate Occ.	473	0%: 0	78.7%: 372	N/A

Overall	22,317	79.3%: 17,699	95%: 21,206	39.3%: 8,773

**Table 3:** Overall and per-dataset match-type percentages and counts, before and after corrections

Match Type	Overall bef. – %: n aft. – %: n	Hospitals bef. – %: n aft. – %: n	Radiation bef. – %: n aft. – %: n	PD bef. – %: n aft. – %: n	Prostate Res. bef. – %: n aft. – %: n	Prostate Occ. bef. – %: n aft. – %: n
Un-matched	20.7: 4,618	0: 0	1: 21	18.4: 3,219	46.6: 905	100: 473
	5: 1,111	0: 0	0.1: 1	2.6: 458	28.3: 551	21.4: 101

Country	0: 0	0: 0	0: 0	0: 0	0: 0	0: 0
	1.5: 337	0: 0	0: 0	1.7: 288	2.3: 44	1.1: 5

State	0: 0	0: 0	0: 0	0: 0	0: 0	0: 0
	2: 462	0: 0	0: 0	2.2: 389	2.5: 49	5.1: 24

County	0.3: 71	0: 0	0: 0	0.3: 47	1.2: 24	0: 0
	2: 446	0: 0	0: 0	2.2: 384	2.9: 57	1.1: 5

MCD	1.7: 381	0: 0	0.3: 5	1.9: 333	2.2: 43	0: 0
	0: 2	0: 0	0: 0	0: 2	0: 0	0: 0

City	49.1: 10,959	4.8: 20	21.2: 426	55.9: 9,763	38.6: 750	0: 0
	28.2: 6,284	0: 0	3.5: 71	31.4: 5,489	25.2: 490	49.5: 234

Zip	4.6: 1,031	7.4: 31	31.7: 638	2.1: 362	0: 0	0: 0
	0.9: 200	0: 0	1.7: 34	0.9: 159	0.3: 6	0.2: 10

Street centroid	0: 0	0: 0	0: 0	0: 0	0: 0	0: 0
	10.8: 2,406	0: 0	1.5: 30	12.1: 2,106	13.4: 261	1.9: 9

Intersection	0: 0	0: 0	0: 0	0: 0	0: 0	0: 0
	9.8: 2,188	0: 0	0.1: 1	12: 2,097	4.3: 83	1.5: 7

Address range	23.2: 5,149	83.5: 349	41.3: 831	21.5: 3,747	11.4: 222	0: 0
	29.6: 6,606	13.4: 56	1.9: 39	34.5: 6,032	20.3: 395	17.8: 84

Nearest parcel	.5: 108	4.3: 18	4.5: 90	0: 0	0: 0	0: 0
	0: 7	0.7: 3	0: 0	0: 2	0.1: 2	0: 0

Exact parcel	0: 0	0: 0	0: 0	0: 0	0: 0	0: 0
	0: 7	0: 0	0: 0	0: 7	0: 0	0: 0

Building centroid	0: 0	0: 0	0: 0	0: 0	0: 0	0: 0
	10.13: 2,261	85.9: 359	91.3: 1,835	0.3: 58	0.3: 6	0.6: 3

bef. = before processing percentages and counts; aft. = after processing percentages and counts

**Table 4:** Match rate improvement contributions per match type with counts and time (sec)

New Type	Overall n t avg t min: max: std	PD n t avg t min: max: std	Prostate Res. n t avg t min: max: std	Prostate Occ. n t avg t min: max: std
Country	320	271	44	5
	98.7	107.9	44.1	79.2
	3: 988: 31,583	3: 988: 29,245	6: 131: 1,942	15: 178: 396

State	455	382	49	24
	120.2	118.4	157	74.7
	3: 1,492: 54,701	5: 1,105: 45,217	3: 1,492: 7,691	19: 210: 1,793

County	295	250	40	5
	90.4	93.4	76.8	48
	6: 889: 26,653	6: 889: 23,342	7: 616: 3,071	32: 86: 240

City	1,405	1,033	138	234
	113.6	108.6	92.8	147.9
	2: 2,287: 159,569	5: 975: 112,176	2: 511: 12,795	9: 2,287: 34,598

Zip	46	45		1
	277.3	249.2	N/A	1,541
	11: 1,541: 12,756	11: 797: 11,215		1,541: 1,541: 1,541

Intersection	173	159	7	7
	110	95.2	200	357.6
	6: 1,063: 19,033	6: 620: 15,130	24: 978: 1,400	32: 1,063: 2,503

Street centroid	345	296	39	9
	127.8	127.5	112.2	190
	4: 1,074: 44,094	4: 1,074: 37,735	5: 629: 4,375	20: 701: 1,710

Address range	430	309	37	84
	106.2	85.2	73.1	198.1
	5: 1,211: 45,670	5: 877: 26,324	6: 314: 2,705	15: 1,211: 16,641

Nearest parcel	2	2		
	449	449	N/A	N/A
	99: 799: 898	99: 799: 898		

Exact parcel	6	6		
	350	350	N/A	N/A
	103: 662: 2,100	103: 662: 2,100		

Building centroid	30	8		3
	279.3	374.6	N/A	208
	41: 816: 8,378	41: 803: 2,997		47: 463: 624

t = time taken (sec) for processing

### Level of accuracy improvements (criteria 2 improvements)

The geocodes associated with 8,773 (39%) of the records were successfully improved as defined in the Methods. The number of low quality geocodes (those associated with large areas), was significantly reduced: city and USPS ZIP code accuracy geocodes were reduced from 10,959 and 1,031 to 6,284 and 200, respectively. The number of building centroid accuracy geocodes (the highest quality geocodes) increased from 0 to 2,261 (10%).

The processing time for the overall dataset and the processing time and spatial shift for the corrected records are listed in Table 5. The distribution of original and corresponding improved match types for the overall dataset including the time taken and spatial shift between the original and improved geocode are listed in Table 6 and Table 7, respectively.

**Table 5:** Processing time for overall dataset and processing time (sec) and spatial shift (km) for improved records only

Dataset	Overall Processing Time (s)	Improved Records Only Processing Time (s)	Improved Records Only Spatial shift (km)
			
	t total t avg t min t max t std	t total t avg t min t max t std	d avg d min d max d std
Hospitals	30,498	26,733	.5
	73	74.3	0
	7	7	6.6
	1,524	1,524	1
	136.3	143.16	

Radiation	403,264	369,799	5.54
	200.5	196.2	0
	5	5	666.2
	4,511	4,511	45.7
	136.3	256	

PD	922,355	634,510	11.6
	52.8	71.9	0
	0	1	6,398.8
	4,717	4,717	109.4
	110.57	127.9	

Prostate Res.	117,969	72,380	12.2
	60.7	86.4	.1
	0	2	870
	4,244	4,244	58
	157.5	193.2	

Prostate Occ.	65,504	60,046	
	138.5	161.4	
	5	9	N/A
	2,287	2,287	
	221	233.3	

Overall	1,538,681	1,163,468	9.9
	69	94.8	0
	0	1	6,398.8
	4,717	4,717	94.4
	145.5	169.4	

t = time taken (sec) for processing; d = distance (km) offset between corrected and uncorrected geocodes

**Table 6:** Match type improvements per original\corrected combination with count and time taken (sec)

New match type\Original match type	County n t avg t min t max t sum	MCD n t avg t min t max t sum	City n t avg t min t max t sum	ZIP n t avg t min t max t sum	Address range n t avg t min t max t sum	Nearest parcel n t avg t min t max t sum
City	32	193	N/A	N/A	N/A	N/A
	51	60				
	6	3				
	573	528				
	1,631	11,583				

ZIP	N/A	N/A	99	N/A	N/A	N/A
			131.1			
			4			
			4,244			
			12,982			

Intersection	6	13	1,975	20	N/A	N/A
	102	40.3	55	81.7		
	17	7	1	13		
	367	136	4,717	878		
	612	524	108,556	1,634		

Street centroid	10	47	1,908	62	N/A	N/A
	94.2	83.3	59.4	148.6		
	24	6	3	6		
	232	379	963	866		
	942	3,916	113,298	9,214		

Address range	6	35	1,868	265	N/A	N/A
	107	104.2	46	66.9		
	25	6	1	6		
	311	649	1,097	867		
	642	3,646	86,007	17,724		

Nearest parcel	N/A	N/A	2	N/A	N/A	N/A
			235.5			
			33			
			438			
			471			

Exact parcel	N/A	N/A	1	N/A	N/A	N/A
			397			
			397			
			397			
			397			

Building centroid	N/A	13	415	591	1,108	104
		127.6	190.8	175.2	174.6	61.9
		23	10	7	5	6
		508	1,524	999	4,511	349
		1,659	79,174	103,530	193,456	6,435

t = time taken (sec) for processing

**Table 7:** Match type improvements per original\corrected combination with count and spatial shift (km)

New match type\Original match type	County n d avg d min d max	MCD n d avg d min d max	City n d avg d min d max	ZIP n d avg d min d max	Address range n d avg d min d max	Nearest parcel n d avg d min d max
City	32	193	N/A	N/A	N/A	N/A
	40.8	9.3				
	0.9	0.1				
	620.3	342.3				

ZIP	N/A	N/A	99	N/A	N/A	N/A
			8.8			
			0.3			
			200.1			

Intersection	6	13	1,975	20	N/A	N/A
	13.4	57.1	10.2	3.6		
	4.2	1.5	0.1	1.2		
	22.7	686.6	3,849.8	6.5		

Street centroid	10	47	1,908	62	N/A	N/A
	29.6	12.4	12.2	110.8		
	4.1	1	0	0.1		
	222.3	322.74	2,363.6	6,398.8		

Address range	6	35	1,868	265	N/A	N/A
	84.2	32.7	9.6	3.8		
	4.4	0.3	0	0		
	239.1	428.2	731.6	206.9		

Nearest parcel	N/A	N/A	2	N/A	N/A	N/A
			20			
			13.7			
			26.2			

Exact parcel	N/A	N/A	1	N/A	N/A	N/A
			3.5			
			3.5			
			3.5			

Building centroid	N/A	13	415	591	1,108	104
		81.9	18.8	2.3	0.1	0
		0.6	0.1	0.1	0	0
		666.2	664.4	56.4	8.1	0.7

d = distance (km) offset between corrected and uncorrected geocodes

### Hospitals dataset

The hospital dataset (n = 418) corrections were completed in 85 hours, averaging 73 seconds per record overall, and 74 seconds per improved record. The match rate for this dataset remained the same (100%), but the majority of the geocodes (294, 70%) had their accuracy increased from address range to building centroid accuracy. The average spatial shift between original and improved geocode location was 450 m.

### Radiation treatment centers dataset

The radiation treatment centers dataset (n = 2,011) correction were completed in three weeks, averaging 201 seconds per record overall, and 196 seconds per improved record. The match rate increased slightly (20 cases, 1%) and 1,865 records (92.7%) had their accuracy levels improved from some lower level to building centroid accuracy. The average spatial shift between original and improved geocode location was 5.54 km.

### Parkinson's Disease (PD) case-control study dataset

The PD dataset (n = 17,471) corrections were completed in 8.6 weeks, averaging 52 seconds per record overall, and 72 seconds per improved record. The match rate increased from 82 to 93% and the geocode accuracy was improved for 6,064 records (35%). The highest rates were for improvements resulting in street centroid, street intersection, and address range accuracy (12.1, 12, and 34.5% respectively). The number of city and USPS ZIP code accuracy geocodes were reduced from 55 and 31% to 2 and less than 1%, respectively. The average spatial shift between initial and improved geocode locations was 11.6 km.

### Residential addresses from prostate cancer case-control study dataset

The residential dataset (n = 1,944) corrections were completed in 1.2 weeks, averaging 60.7 seconds per record overall, and 86.4 seconds per improved record. The match rate increased from 53 to 72% and the highest rates of improvements were to the street centroid and address range level accuracies (13.4 and 20.3%, respectively). City level accuracy geocodes were reduced from 38.6 to 25.2%. The average spatial shift between initial and improved geocode locations was 12.2 km.

### Occupational addresses from prostate cancer case-control study dataset

The occupational dataset (n = 473) corrections were completed in 0.5 weeks, averaging 138.5 seconds per record overall, 161 seconds per improved record. The match rate increased from 0 to 79% and the highest rates of improvements were to city and address range level accuracy (49.5 and 17.8%, respectively). There were no spatial shifts because none of the original geocodes had an initial location from which this measure could be determined.

## Methods

### Study Data Sources

The data used for this study consisted of address data from five different sources. Three were from ongoing epidemiological studies of risk factors for disease: 17,471 addresses from a case-control study of Parkinson's Disease (PD) (cases had been diagnosed with PD, and control subjects were recruited using mailings based on tax-assessor parcel records); 2,417 addresses from a case-control study of prostate cancer, where cases came from the population-based cancer registry, and controls also came from tax-assessor record-based mailings; these subjects provided both residential and occupational historical datasets containing 1,944 and 473 addresses, respectively. In both the PD and prostate cancer studies, the purpose of obtaining geocodable data was to determine proximity to areas of pesticide and other environmental exposures, so obtaining accurate geocodes was essential. In both studies, participants were interviewed to obtain lifetime residential histories, and in the prostate study, lifetime occupational address histories as well. These data are from individuals living in Kern, Tulare, and Fresno counties within the State of California, representing the full spectrum of the rural-urban continuum, as well as areas with varying population densities and mixes of residential and commercial locations.

The two remaining data sources were address lists of 418 hospitals and 2,011 radiation treatment centers in the State of California. These were being geocoded for use in a variety of studies assessing the effect of distance to service provider on health outcomes (e.g. the impact on later stage of cancer at diagnosis of living further away from a radiation treatment center) and would also be useful for such tasks as emergency routing. The hospitals and radiation treatment centers and their addresses were obtained from a comprehensive state-wide listing used for hospital registration purposes [44].

The hospital data are representative of large commercial health care facilities. These locations typically consist of one or more large buildings (and associated parking lots) on large campuses and/or parcels of land. The radiation treatment center data are representative of small commercial health facilities. These locations range from large standalone treatment facilities similar to (or contained within) the large hospitals in the Hospitals dataset to small retail-type offices located in standalone buildings or commercial strip malls.

Overall, these data sources are representative of those used in many health GIS studies. The data from the case-control studies are typical of those used in epidemiological studies of disease risk factors, where participants have the opportunity to describe their residential locations. By contrast, the hospital and radiation facility data come 'as is', but are useful as proxies for geographical measures of access to care [45-47].

### Study Data Quality

The address information contained within each of the datasets used for this study varied in quality. For this study we defined the quality of the address data within a dataset in terms of how well the whole set of data could be geocoded. We defined "very good quality" to be locational data for each record corresponding to postal address data that included the standard postal address fields such as street address, city, state, and United States Postal Service (USPS) ZIP code, e.g., "3620 S. Vermont Ave, Los Angeles, CA, 90089". If the aforementioned components of the street address were parsed into their separate components (with "3620", "S.", "Vermont", "Ave" each representing the number, pre-directional, name, and suffix portions of the postal street address, respectively), the assessment was upgraded to "excellent".

The quality of the data was considered "good" when the information contained in the street address portion of each record included non-postal address data such as named facilities, e.g., "Cardinal Gardens", county names, and relative locational descriptions, e.g., "just down the road from Exposition Park". The assessment was downgraded to "poor" for those cases in which data transpositions occurred, data elements were omitted, and/or completely invalid data began to appear in place of an address. The data was considered "very poor" when the address data were not separated into sub-components and some/most of the locational data did not describe the locations at all.

Each of the datasets was characterized as one of these classes based on the addresses it contained. The quality of the hospital and radiation treatment center address data can be characterized as very good because all records contain a full (non-parsed) postal street address, city, state, and USPS ZIP code. The quality of the PD address data source can be characterized as poor because although 64% of the records include data in the street address fields, the address data in these fields are quite poor. The quality of the prostate residential address data can be characterized as fair because over half (56%) of the data include a full street address, and the quality of these address data are also fair. The prostate occupational data can be characterized as very poor because none of the records contained any information in any of the address fields; instead they contain a single attribute called "AddrNotes" which can best be described as a free-text description of where the individual was employed, with roughly 70% of this pertaining to an address in the form of a just business name and city.

### Study Batch Geocoding Engine

All records were initially geocoded with a geocoding engine built and maintained by the USC GIS Research Laboratory. This service is hosted at USC [43] and is freely available to any researcher who wishes to use it. The exact details of the geocoder configuration can be found on the USC site [43], and we will only provide a brief overview of the main components here.

The implementation of the geocoder used for this study performed strictly deterministic feature matching with attribute relaxation using the following reference data sources: 2005 US Census Bureau TIGER/Line files [48], US Census Bureau Cartographic Boundary Files [49] including the County, Minor Civil Division, Place, and Zip Code Tabulation Area (ZCTA) layers, and Los Angeles County Assessor's parcel boundaries (LA Assessor Files) [50]. The linear interpolation techniques described in [51] were used for the TIGER/Line street segments, while Green's Theorem was used to obtain the geometric center of parcels and the geographic centroids from the US Census Bureau Cartographic Boundary File features (County, etc.) were used directly. A non-USPS CASS-certified address parser was used to identify the components of an input address. Because of the advanced feature matching, feature interpolation, and additional reference data sources implemented in the USC geocoder, the possible geocode qualities used in this study are based on an augmented version of the NAACCR GIS Coordinate Quality Codes [52], shown in Table 1.

### Manual intervention and re-geocoding interface

**Figure 1:**
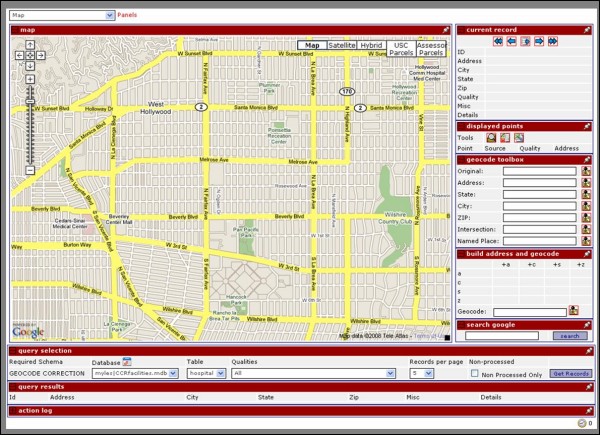
Manual intervention interface.

As part of this study a manual intervention and re-geocoding interface was developed as a web-based application. This interface is hosted at USC [43] and is freely available to all researchers who wish to use it. The service allows a user to upload and interactively process a database of geocoded records in batch, securely over a 128-bit encrypted secure socket layer (SSL) connection in their web browser. The main interface, shown in Figure 1, consists of map, displayed points, record navigation, record selection, and re-geocoding panels. The map panel utilized is an implementation of the Google Maps API, as is the geocoder used for re-geocoding [53]. Technical details on how the Google Maps API can be used to display data are available in [54] as well as from the Google web site [53]. According to the online documentation, the Google Maps API geocoder is based on the Tele Atlas US road network [55]. Other than this detail, little documentation is available describing the components of the Google Maps API geocoder, although there is a substantial amount of user discussion in online newsgroups that suggests it is a probabilistic matching system.

To process a database of geocoded records, a user first uploads a database and maps the fields in their database to the fields the service is expecting. Next, the user selects the type of geocode qualities they wish to work on (or alternatively selects all records), and clicks the "get records" button to display the first set of records from the database. The user can navigate backwards and forwards through the page of records displayed or through different pages of records within the database using the navigation panel. When a record is actively being displayed, the original geocoded point associated with it is displayed on the map. The user can utilize the built in Google Maps functionalities, including zooming, panning, and the selection of several different data layers to view, e.g., satellite/aerial imagery, street networks, or terrain models.

**Figure 2:**
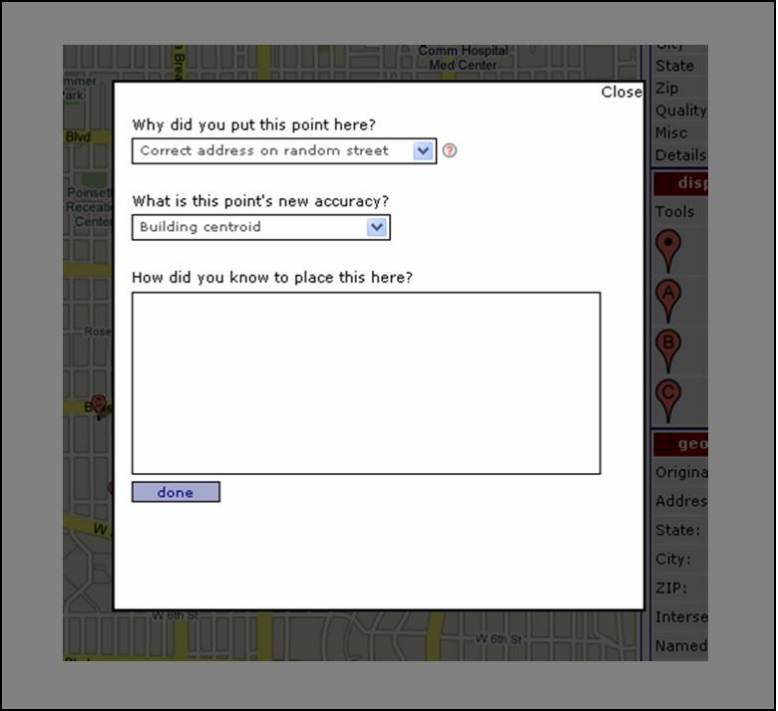
Prompt for new accuracy description and placement rationale.

The correction of a geocode creates another point on the map using one or more of the available options; clicking a location on the map, dragging an existing point, or entering information into one of the geocode boxes and trying to re-geocode it. Performing any one of these options creates a new point, adds it to the panel of displayed points, and adds it to the map. When a point is created by either clicking on the map or dragging an existing point to a new location, the user is prompted to indicate the new accuracy level and provide a rationale for how and why they placed the point where they did, as shown in Figure 2.

**Figure 3:**
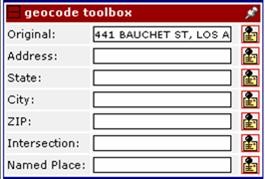
Re-geocoding data entry options.

**Figure 4:**
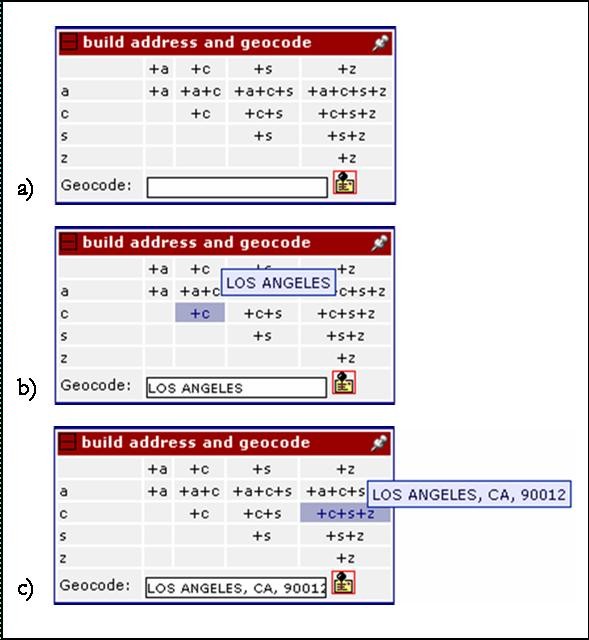
**Address creation matrix**. a) no field selection, b) city field selection, c) city, state, USPS ZIP combination field selection.

If the new point is created by re-geocoding using the Google Maps geocoder, the system records the level of accuracy returned from the Google Maps API as a result. The Google Maps geocoder panels are separated into several input fields to obtain information about which portion of the address the user attempted to re-geocode, as shown in Figure 3. When a record is selected for processing, the original address is automatically filled in the "original address" field, so that the user can easily submit it for simple re-geocoding as fast as possible. Alternatively, if the user determines that the original address is something other than a street address, they can enter it into one of the other fields such that the system can keep track of the type of information they attempted to re-geocode, e.g., street intersection, named place, etc. The user can also build an address for re-geocoding from the components of the complete address associated with the record (street address, city, state, USPS Zip code) by clicking on the address matrix portion of the re-geocoding panel as shown in Figure 4. Using this, the user can quickly select individual and/or different combinations of the original address data for re-geocoding. This is useful in the case of transposed address components and/or extraneous data that need not be included in the re-geocoding query, enabling the user to easily re-submit portions of the address for re-geocoding without the need to type anything, again increasing the speed with which they can work on their datasets. If the information they attempt to re-geocode using the Google Maps geocoder returns multiple ambiguous matches, they are all added to the displayed points and map panels.

After creating one or more new points the user chooses and selects the "corrected" one and stores it in the database to be associated with the record. This stores the new point and all relevant metadata about its creation with the record in the database. The correction process is repeated for each record in the database until each of the records that warranted checking has been processed. Two sets of records were skipped altogether: those for which the quality of the geocode was sufficiently high to begin with that no correction was needed and those for which correction was not possible.

### Manual intervention and re-geocoding protocol

The users of the system were directed to always use the same protocol when attempting to correct and/or re-geocode an input record. Each user was assigned to work on a different level of original geocode accuracy, with multiple users being assigned the same one in some cases. The geocodes with the lowest accuracy were attempted first. For each record, the users were instructed to first attempt to simply re-geocode the original address. If this was successful in improving the accuracy to a sufficient level, they would store the re-geocoded point as the corrected one. If not, the user would then perform background research using online searches based on information associated with the record to determine what a true corrected address should be. These searches are also performed within the interface so the queries a user performed to obtain more information are also associated as metadata with the corrected record.

The goal was to geocode every record to building centroid accuracy. If the correct building could be unambiguously determined either through a subjective process, e.g., there was only a single building on the street, or through evidence found online, e.g., pictures or descriptions of locations, a corrected geocode was placed at the centroid of the roof centerline, either by clicking on the map or dragging an existing point. The users were directed to place the point as close to the centroid of the building as they could, no matter the size of the building. Any information used to determine this location (i.e., the rationale) was then recorded when the user was prompted to indicate the new geocode's accuracy. If an exact building centroid could not be determined, the users were instructed to attempt to obtain geocodes following the hierarchy defined in Table 1.

### Study user group

The user group who participated in this study and worked with the interactive tool to perform the manual intervention consisted of four full-time paid staff members, and three volunteer graduate students. The quality of the work was assessed and verified collectively by two of the authors of this report (DG and MC) by randomly checking 1% of the processed results both during and after the study. This verification consisted of: 1) visually inspecting the placement of the resulting geocode location; 2) a logical check to determine that the correct process and protocol were followed in the determination of how and why the geocode was placed where it was in the case of a successful correction; and 3) a logical check that non-correctable records were in fact non-correctable.

## Discussion

### Main findings

The geocode correction approach used for this research can be used to substantially improve both the match rate and quality of individual geocodes with a relatively small outlay of time and effort. The process was also capable of improving large and small scale datasets as well as those with high and low initial quality. Much of the gain in improvement for the largest PD dataset which contained 17, 471 records, was achieved because the geocode correction users were able to re-geocode the original address using the Google Maps geocoder which was capable of achieving street centroid and street intersection level accuracy. By contrast, the original USC geocoder reverted to the next least uncertain geocode, in many cases either the city or ZCTA centroid. The hospital and radiation treatment centers, which would have been considered the most accurate dataset because they had the highest initial match rates, were substantially improved in terms of the level of accuracy associated with their geocodes, resulting in 86 and 91% building level accuracy at an average cost of 74.4 and 193.3 seconds per record, respectively. Our 16% improvement in overall match rate is slightly better than the 10% increase found in [31] using an interactive manual review approach.

The characteristics of the datasets (described in the Methods) had a substantial effect on the quality of results achieved with both the USC geocoding platform and geocode correction interface. For instance, the Hospitals dataset had high initial quality with a 100% match rate and 83% of the geocodes having address range accuracy. After applying the manual intervention process, 86% of the geocodes were improved to building centroid accuracy, averaging 73 seconds per record. This result indicates that datasets with very good quality initial address data can be improved to extremely high accuracies at a reasonable pace. Likewise, the initial geocoding on the Radiation treatment centers dataset was also able to achieve a high match rate (99%), but the majority of initial match types were to the city and USPS ZIP code levels (21 and 32%, respectively). The vast majority (~85%) of these records were geocoded to building centroid accuracy after correction.

Both of the aforementioned datasets represent business locations, so one reason why these high levels of accuracy were achievable might be that web-based information was readily available and provided a proxy source of data for correcting the geocode, which is not as readily available for residential addresses. Alternatively, the Occupational dataset, which is described in the Methods section as being of very poor quality, was entirely non-geocodable by the USC geocoding platform because the input address data were not parsed, and were essentially free-text. After correction processing, although the match rate increased to 79%, the accuracy of roughly half of the records (49%) remained quite low, only achieving city centroid accuracy. These addresses also represent businesses, but the low overall resulting accuracy for this dataset might be due to the fact that they are not well reported in terms of address accuracy, and specifically not reported by people who use them for geocoding. In contrast, the hospitals and radiation treatment centers have been previously used to identify the actual location of cancer diagnosis, etc., and accordingly must be matchable to an actual location – not necessarily to geocode, but certainly to send bill payments.

We have reported the spatial shift in all cases where the geocode was moved from one location to another, regardless of the initial geocode accuracy. One could argue that it is a meaningless measure if the original geocode was clearly worthless. If we assume most studies would heed the warnings in the literature [e.g., [56]] and be hesitant to simply exclude records with low level accuracies, these low-level geocodes could potentially be used in studies. Therefore it is important to report on how large these spatial differences can be in order to demonstrate that low quality geocodes should not be used directly; instead they should be corrected in some fashion before they are used (e.g., the approach presented herein, geo-imputation). Our results do in fact show that the distances between the original geocodes and the corrected geocodes are quite substantial across all of the datasets, averaging 9 km for all five datasets combined. It is expected that a change in distance of this size would have an effect on the accuracy of studies using USPS ZIP code size or smaller units of analysis. Most studies that use geocoded data to assign an "exposure" use buffers much smaller than 9 km, e.g., 500 or 1,000 m [57], so starting with an error of 9 km renders these results essentially useless. This large average distance is in many cases caused by the differences between the US Census Bureau geographic base file centroids used by the USC geocoder [43] and (presumably) the commercial alternatives from Tele Atlas used by the Google Maps geocoder [53]. The variability in these distances is substantial, ranging from less than 1 m in the case of corrections from the nearest parcel to building centroids, to well over 6,000 km in the case of USPS ZIP code level accuracies where the wrong USPS ZIP codes are corrected to address range level accuracies, with average distances per dataset ranging from 450 m to 12.18 km.

The variations in the spatial shifts between the datasets are also partially explained by the characteristics of the addresses in the datasets. The extremely large differences, which skew the overall average, were typically caused by the USC geocoder not being able to geocode street centroids and street intersections and reverting to lower accuracy geocodes, while the Google Maps geocoder could output the higher accuracy locations. Therefore, the datasets with the higher percentages of intersection and street centroid accuracy address data show a higher percentage of improvements resulting in large spatial shifts after manual intervention. Alternatively, the datasets with a higher prevalence of postal address data (hospitals and radiation treatment centers) were correctly processed by the USC geocoder to an initially high level of accuracy, resulting in corrections with smaller spatial shifts following manual intervention.

An examination of the data revealed that the USPS ZIP code to address range correction overall average distance of 110.8 km (from Table 6) mainly derives from instances where a single digit in the last two or three digits of the original USPS ZIP code value was incorrect. In these cases the USC geocoder would fail to match to a street-level geocode and would revert to the centroid of the (incorrect) USPS ZIP code value in the record, which would typically be within a 100 km region nearby where the correct address should be because of the inherent geographic distribution of USPS ZIP code values that are close to each other. Further research needs to be conducted to determine if this is indicative of systematic problems similar to that identified by Boscoe et al. [33].

Our average processing time of 69 seconds per record was consistent with the findings of Davis Jr. [41], but was less than both the 3 and 3–6 minute per record times reported by Boscoe et al. [33] and Strickland et al. [11], respectively. However, like Strickland et al. [11] we found that the characteristics of a dataset also have a substantial effect on the amount of effort (time) required to process it. The geocode correction process generated building level accuracies for many of the records in the Hospital and Radiation treatment centers datasets; however, the average time to process a record in these datasets was 73 and 200 seconds, respectively. One explanation for this difference is that hospitals tend to be large buildings, occupying large areas, while the radiation facilities tend to be located within business parks and/or strip malls. The large structures that make up a hospital are often clearly identifiable from the satellite/aerial imagery view in the geocode correction interface, with some being labeled as part of the Google Maps display, making it quick and easy to identify the corrected location to associate with the record. Conversely, the individual performing the correction was more likely to have to search online to determine which structure among many small ones the radiation facility resides in to determine the correct location of the radiation facility. Those using the geocode correction interface reported going the additional step to look for the location within the building and/or strip mall on images available on the Internet to specify the centroid of the building in many of these instances. This undoubtedly increased the per record processing time in these cases.

In addition to the differences in effort (time) expended to improve the two aforementioned datasets, we also see that the time required to correct the very poor dataset was also quite high. One explanation for this is that because the address data associated with each record were so poor, typically only including a business name and city/state and/or city/county pair, the individuals performing the geocoding correction spent a substantial amount of time searching online to determine the address of the business listed along with the record. They were only able to obtain a geocode for the correct city in many of these cases.

The software development for the manual intervention interface was completed by a single full-time programmer over the course of a six month period. This development represents a substantial initial cost, but once complete has required no ongoing charges other than the correction of small programming bugs. To ensure a greater level of security and confidentiality, a secure socket layer (SSL) certificate was purchased for the site, costing $700 for a three year period. As previously noted, this service is available for free to all researchers who wish to use it, and the complete source code for the interface described herein can be made available, thus lowering the upfront cost to other organizations interested in attempting a similar approach, in-house or over the Internet. The initial training required to familiarize the users with how to use the interface was completed within two hours per user, on average, and is essentially a negligible cost. The majority of the users who participated in this study had little experience with any type of geocoding, but were generally familiar with using an online map interface such as the Google Map API used in this study.

### Limitations

The results presented herein are subject to several limitations. First, we do not have ground truth data for any of the addresses in this study, and the "true locations" used as the geocode corrections are completely reliant on the accuracy of the Google Maps API and data sources. The accuracy of the satellite imagery, road vectors, and geocoding used for the Google Maps API is dependent on the underlying Navteq and Tele Atlas data. However, manual geocoding with aerial/satellite imagery has been proposed as a substitute for ground truth data in the literature [34,42] and has been used as such on numerous occasions [2,6,11,15,18,19,25,28,29,35,36]. Our approach also assumes that the Google Maps API and associated data are available in the region(s) of interest. This is most likely not a serious problem for the US due to the almost complete coverage, but for other countries the underlying data may be limited.

Next, our protocol directed the users to place the corrected location at the centroid of the building in all cases possible, no matter the size of the building. We made this decision for two reasons: 1) choosing the centroid minimizes the maximum error that could be introduced, and 2) we assume that most people spend the majority of their time close to the center of a building. For large buildings, like most hospitals, this choice may have introduced spatial error that could be larger than that introduced if any random location within a building was chosen for a smaller building, like a radiation center in a strip mall. If further information was available as to where within a building (of any size) the correct point should be placed the users would have used it to guide their placement. Without this information, we feel that our protocol is a conservative way to minimize the potential spatial error that could be introduced. Individual researchers may need to weigh the benefits and drawbacks of our centroid placement rationale to decide if this is the correct action for them to take based on their study constraints.

The approach presented herein may not be suitable for all researchers under all conditions. This may be the case for a researcher working with personally identifiable information, where they need to take care that these data remain secure under all circumstances. Accordingly, some organizations may have safeguards in place that prohibit Internet use of any kind while working with these data, which would thus prohibit the use of the web-based service outlined herein. However, our approach could theoretically be applied in an "offline" mode where all of the underlying data were hosted locally within an organization, foregoing the need to use any Internet resources but also requiring that the underlying data be obtained. Another limiting factor with employing our method may be the usage of the Google Maps geocoder. Utilizing a third party service for geocoding and/or viewing maps or a region may or may not be an acceptable practice depending on the rules and regulations of an organization, a researcher, or due to data licensing/use agreements. However, emerging research is beginning to offer guidance for secure record linkage which could be applied to the geocoding process [58].

Our results are undeniably affected by the geocoders used both for the initial geocoding (the USC geocoder) as well as the re-geocoding (the Google API geocoder). These two geocoders had fundamentally different capabilities, mainly due to the time and effort that have been spent on their construction. The USC geocoder is a research platform that was created in-house (as described in the Methods) to test various geocoding strategies, and as such was missing many key capabilities present in the Google API geocoder which was the product of a commercial enterprise (e.g., probabilistic matching, street intersection parsing, and street centroid interpolation) when this study was conducted. These shortcomings certainly led to some number of low accuracy initial geocodes that may have otherwise been high accuracy had a higher quality initial geocoder been used (e.g., the case of all street intersections failing and reverting to a lower-level-accuracy geocode).

Therefore the level of improvement achieved by researchers replicating this study with a different combination of datasets and geocoders may be different than that reported here. It would be informative for other researchers to replicate the present work with more competent (i.e., expensive) geocoding software to determine if the improvement effects observed in the present work are directly translatable, or if our correction methods begin to lose their appeal as more money is spent up-front on the geocoding software instead of after the fact to improve its results. It is anticipated that because much of the work performed on many of the records was background research (as evidenced from the high time per record), using an improved initial geocoder would not have substantially lowered the levels of improvement reported here. We argue that no matter what initial geocoder is used, some level of manual intervention/interactive geocoding will always be required within any geocoded datasets because address data are routinely reported in vague terms and even the highest quality address data will be beholden to the accuracy of the geocoding process used. The frequent use of the manual methods for improving inaccurate geocodes in the available literature seems to support this [2,11,14,15,17-19,26-29,34-36].

Finally, we recognize that the use of the US Census Bureau ZIP Code Tabulation Area (ZCTA) layers in the initial batch geocoding (as described in the Methods) instead of a commercial approximation of the USPS ZIP code delivery areas represents a contentious issue and one that is frequently cited in the literature. While the two layers do not fundamentally represent the same thing (one actual areas and the other delivery routes), we feel this choice is justified because all geocode results having worse than street level accuracy will be corrected as part of this study, and therefore the issue is moot in our specific circumstances. Other studies that skip or ignore these subtleties should read and heed the literature on this topic (see [13] for an in-depth review).

## Conclusion

Manual geocode correction is a feasible and economical method for improving the quality of geocoded data. It results in increased match rates and improvement in the quality of the underlying geocodes. The process can be successfully applied to both initially high and low quality geocodes and their associated underlying address data, and has been shown to work well for both large and small datasets. In particular, the combined strategy of utilizing a freely available, adequately performing geocoder first and then subsequently manually/interactively correcting the geocodes with a web-based interface appears to be a suitable method to achieve very high match rates and fairly high quality geocoded data with a non-trivial but feasible amount of effort.

Both the USC geocoder and USC geocode correction tool utilized in this study are freely accessible to the greater research community through the USC GIS Research Lab website [43]. The complete source code for the geocode correction web service can be made available upon request. The availability of these resources should further help defer the upfront costs one might encounter if they sought to create similar geocode correction tools themselves. It is hoped that other researchers will employ the same or similar methods to ensure that the geocodes they base their research on are of the highest possible quality. Further, the authors echo the growing chorus of researchers e.g., [17] in encouraging all researchers utilizing geocoded data to report quantitative metrics about the geocodes used in their work, such as those presented in this paper, in addition to simply describing their match rates alone.

## Competing interests

The authors declare that they have no competing interests.

## Authors' contributions

DG conceived of and designed the study, developed the USC geocoding engine, developed the geocode correction interface, performed the statistical analysis, and wrote the manuscript. MC participated in the design of the study, coordinated its execution, and provided comments and suggestions on the manuscript. JW, CK, and BR provided comments and suggestions on the manuscript. All authors read and approved the final manuscript.
